# The Voluntary Contributor

**Published:** 1912-07-27

**Authors:** 


### OUR NATIONAL INSURANCE ACT DEPARTMENT.

### XXI.—The Voluntary Contributor.

The National Insurance Act is framed principally for the benefit of employed persons, that is to say, for persons who are working for an employer under a contract of service, whether express or implied. There is, however, another class of persons who may benefit from the Act if they elect to do so. This class is to be known as voluntary contributors, and any man or woman who fulfils the conditions laid down by the Act may become a voluntary contributor. In order to fulfil these conditions the person must be engaged in some regular occupation, and must be mainly or wholly dependent for his or her livelihood on the earnings derived from that occupation, and the total income of such person from all sources must not exceed £160 per annum. After the Act has been in force five years it will be possible for persons to be insured as voluntary contributors who are in receipt of an income exceeding the limit mentioned if they have been insured either as employed contributors or as voluntary contributors for a period of not less than five years. There is one important exception that must be made to this general statement, namely, that a married woman is not entitled to become a voluntary contributor, and if a woman is before marriage a voluntary contributor she is not entitled on marriage to continue to be such a contributor, except at the special rate of contribution for reduced benefits as laid down by the Act.

### Who Can Become a Voluntary Contributor.

From these conditions it will be seen that at the outset the class of voluntary contributors will consist of small tradesmen and others working on their own account who may desire to make provision for sickness or disablement. In the course of time it is likely that the class will be principally composed of those who have been in insurance for a period of at least five years, because the continuation of membership after the rate of remuneration exceeds £160 per annum is the only way in which an insured person can obtain full advantage of the contributions paid on his behalf during the years that he was in insurance whilst receiving remuneration at a rate not exceeding the limit.

### Rates of Contributions.

It is important that all persons who come within the category of those entitled to become voluntary contributors should be aware of the fact that the rate of contribution that they will have to pay depends upon the time at which they exercise their option of becoming insured. The rates differ according as entry into insurance is made before January 15 next, or on or after that date. To voluntary contributors who enter insurance within six months of the commencement of the Act, and whose age at the time of entry is below forty-five, the rate of contribution is the same as the employed rate, that is to say, 7d. a week in the case of men, and 6d. a week in the case of women. Above the age of forty-five the contribution is dependent upon the age, and is in accordance with the scale drawn up by the Insurance Commissioners. These rates are sufficient to cover seven-ninths of the benefits conferred by the Act in the case of men, the balance of two-ninths being provided by the State. In the case of women the contributions are framed to cover three-quarters of the benefits, the balance of one-quarter being provided by the State. It must, of course, be noted that in the case of the voluntary contributor, for the obvious reason that there is no employer, the whole of the contribution has to be paid by the contributor; there is no division of the contribution, as in the case of employed contributors, between employer and employee.

If entry into insurance as a voluntary contributor is deferred until January 15 next or after, the rate of contribution will depend in all cases on the age at entry into insurance, and the table that has been framed by the Commissioners in accordance with the Act does not allow of the State grant towards the benefits conferred. It will thus be seen that it is manifestly to the advantage of a person who is entitled to become a voluntary contributor to exercise the option within the period allowed. To emphasise this point we reproduce in the following schedule examples of the rates of contributions required for male voluntary contributors in England, Scotland, and Wales, setting forth in parallel columns the rates appropriate to the age last birthday, as stated in the first column according as entry is made (1) before or (2) on or after January 15, 1913. The table is only given for sample ages. The rates vary from age to age, and full information can be obtained, if desired, on application at a post office or from any approved society.

Rates of Contributions for Male Voluntary Contributors.
England, Scotland and Wales.

| Age last Birthday | Weekly Contribution | |
|---|---|---|
| | Entry before January 15, 1913 | Entry on or after January 15, 1913 |
| | s. d. | s. d. |
| 16 ... ... | 0 7 | 0 7 |
| 20 ... ... | 0 7 | 0 7½ |
| 25 ... ... | 0 7 | 0 8 |
| 30 ... ... | 0 7 | 0 8 |
| 35 ... ... | 0 7 | 0 9 |
| 40 ... ... | 0 7 | 0 9½ |
| 45 ... ... | 0 9 | 0 10½ |
| 50 ... ... | 0 10½ | 1 0 |
| 55 ... ... | 1 0½ | 1 2 |
| 60 ... ... | 1 2½ | 1 4½ |

Entry may be made up to the age of sixty-five, and the contributions cease at the age of seventy. The rate of contribution for male voluntary contributors in Ireland ranges from 2d. to 3d. a week less than the figures above mentioned, as it will be remembered that the benefits conferred in Ireland do not include medical benefit. In a similar way the contributions for women are less than those quoted, the difference ranging from 1d. a week at the younger ages to 2d. a week at the highest ages. This is again due to the fact that the benefits are on a lower scale for women than for men.

Advantages of Joining before January 15, 1913.

In spite of the opposition to the Act we are informed that many persons have already availed themselves of the provisions of the Act by joining approved societies as voluntary contributors. There are many advantages in this course of action, particularly if the age at entry is less than forty-five. Up to the age of forty-five, if entry into insurance is made within the first six months after the Act comes into operation, as we have already pointed out, the weekly contribution is 7d. for men and 6d. for women. These rates strictly apply to those who enter insurance at the age of sixteen last birthday, and for all persons entering at more advanced ages the contribution should theoretically be at a higher rate. This is so for two reasons. In the first place, sickness rates increase with age, and the higher the age the greater is the liability to sickness, so that the period when the greatest demand will be made for sickness benefits is much nearer in the case of a person entering insurance at the age of forty, say, than for a person entering at the age of sixteen. In the second place, the length of time during which contributions can be paid is much less. The result, therefore, of allowing persons to enter insurance at ages above sixteen, whilst paying only the rate of contribution applicable to that age, is to incur a liability greater than the contributions will bear. In order to provide for this liability the necessary reserves are being set up by the State. For voluntary contributors in England, Scotland, and Wales the amounts of these reserves are at the age of seventeen 9s., at the age of twenty £1 15s., at the age of thirty £4 13s., at the age of forty £7 9s., and at the age of forty-four £8 16s.

It is thus seen that very substantial advantages are to be gained by those persons who enter insurance now at ages below forty-five, and it is therefore important that all who are entitled to avail themselves of these advantages should not allow the opportunity to pass without giving the matter due consideration.

A person who enters insurance as a voluntary contributor may not always remain in that category. He may become employed within the meaning of that term as defined in the Act, and in this event he will be transferred to the class of employed contributors. His employer will consequently be required to pay the employer's portion of the weekly contribution, but the total contribution will remain unaltered unless the insured person gives notice of his wish to be transferred to the employed rate. If such notice is given the rate payable in respect of him will be the employed rate, but the rate of sickness benefit payable in respect of him will be reduced as though he had not previously been insured, subject to such addition as represents the value at that time of the contributions previously paid by him. In a similar way, a person who enters insurance as an employed contributor may be transferred to the voluntary class.

It has already been pointed out, indirectly, that when such change occurs after the person has been insured for at least five years he does not suffer in consequence. If, however, the change is made within five years from entry into insurance he will be deemed to be in arrear, as from the date when he so became a voluntary contributor, to the amount of the difference between the aggregate contributions paid since entry into insurance and the aggregate contributions that would have been paid had he throughout been a voluntary contributor. He will also lose the benefit of the difference, if any, between the amount of the reserve that has been credited to his society on his behalf and the reserve that would have been credited to that society if he had originally become a voluntary contributor.

The Benefits Receivable.

The benefits provided for voluntary contributors are the same as those for employed contributors with two exceptions. In the first place, it will be remembered that sickness benefit as provided for employed contributors entering insurance at ages over fifty and under sixty is at the reduced rate of 7s. a week in the case of men and 6s. a week in the case of women, and this benefit is still further reduced when the age at entry is over sixty. It is clear from the scale of contributions prescribed by the Commissioners that no such reduction is to be imposed in the case of voluntary contributors, but that in all cases the sickness benefit will be at the rate of 10s. a week for men and 7s. 6d. a week for women, subject only to modification when the contributor is in arrear and to reduction in the case of unmarried minors. In the second place, there is a modification in the case of maternity benefit in regard to the time that must elapse after entry before the insured person is entitled to the benefit. In the case of the voluntary contributor fifty-two weeks must have elapsed since entry into insurance, and at least fifty-two contributions must have been paid before maternity benefit can be claimed. The number of weeks and the number of contributions in the case of an employed contributor is twenty-six. The reduction of benefits when the voluntary contributor is in arrear is to be governed by a table prescribed by the Insurance Commissioners, but so far as we are aware this table has not yet been published. The privilege accorded to employed contributors of ignoring any arrears of contribution that may have fallen due in the year after the Act came into operation does not apply to voluntary contributors.

[We regret having had to hold over the article on sanatorium benefit we hoped to publish this week. Ed. The Hospital.]

NOTE.—Questions and Correspondence on all doubtful points are invited, and should be addressed to the Editor, The Hospital, 28 Southampton Street, Strand, W.C., and marked "Insurance."

